# A combination of trastuzumab and BAG-1 inhibition synergistically targets HER2 positive breast cancer cells

**DOI:** 10.18632/oncotarget.7944

**Published:** 2016-03-06

**Authors:** Emmanouil Papadakis, Natalia Robson, Alison Yeomans, Sarah Bailey, Stephanie Laversin, Stephen Beers, A. Emre Sayan, Margaret Ashton-Key, Stefan Schwaiger, Hermann Stuppner, Jakob Troppmair, Graham Packham, Ramsey Cutress

**Affiliations:** ^1^ Cancer Research UK Centre Cancer Sciences Unit, Southampton General Hospital, Southampton, United Kingdom; ^2^ University Hospital Southampton, University of Southampton Faculty of Medicine, Southampton General Hospital, Southampton, United Kingdom; ^3^ Institute of Pharmacy/Pharmacognosy, Center of Molecular Biosciences, University of Innsbruck, Innsbruck, Austria; ^4^ Daniel Swarovski Research Laboratory, Department of Visceral, Transplant and Thoracic Surgery, Innsbruck Medical University, Innsbruck, Austria

**Keywords:** breast cancer, BAG-1, HER2, trastuzumab, resistance

## Abstract

Treatment of HER2+ breast cancer with trastuzumab is effective and combination anti-HER2 therapies have demonstrated benefit over monotherapy in the neoadjuvant and metastatic settings. This study investigated the therapeutic potential of targeting the BAG-1 protein co-chaperone in trastuzumab-responsive or -resistant cells. In the METABRIC dataset, BAG-1 mRNA was significantly elevated in HER2+ breast tumors and predicted overall survival in a multivariate analysis (HR = 0.81; *p* = 0.022). In a breast cell line panel, BAG-1 protein was increased in HER2+ cells and was required for optimal growth as shown by siRNA knockdown. Overexpression of BAG-1S in HER2+ SKBR3 cells blocked growth inhibition by trastuzumab, whereas overexpression of a mutant BAG-1S protein (BAG-1S H3AB), defective in binding HSC70, potentiated the effect of trastuzumab. Injection of a Tet-On SKBR3 clone, induced to overexpress myc-BAG-1S into the mammary fat pads of immunocompromised mice, resulted in 2-fold larger tumors compared to uninduced controls. Induction of myc-BAG-1S expression in two Tet-On SKBR3 clones attenuated growth inhibition by trastuzumab *in vitro.* Targeting endogenous BAG-1 by siRNA enhanced growth inhibition of SKBR3 and BT474 cells by trastuzumab, while BAG-1 protein-protein interaction inhibitor (Thio-S or Thio-2) plus trastuzumab combination treatment synergistically attenuated growth. In BT474 cells this reduced protein synthesis, caused G1/S cell cycle arrest and targeted the ERK and AKT signaling pathways. In a SKBR3 subpopulation with acquired resistance to trastuzumab BAG-1 targeting remained effective and either Thio-2 or BAG-1 siRNA reduced growth more compared to trastuzumab-responsive parental cells. In summary, targeting BAG-1 function in combination with anti-HER2 therapy might prove beneficial.

## INTRODUCTION

Amplification of the human epidermal growth factor receptor 2 (HER2) gene occurs in 15%–30% of breast cancers and results in high levels of HER2 protein expression [1]. This is accompanied by increased HER2 signaling and promotes malignant cell growth and survival [2]. Therefore, patients whose tumors are characterized by HER2 gene amplification and protein overexpression develop a more aggressive type of cancer, which is associated with poor prognosis [3]. The humanized antibody trastuzumab, which targets the extracellular domain of the HER2 receptor, can prolong overall survival when used as a single agent [4] or when combined with chemotherapy [5]. However, 74% of patients with HER2+ metastatic breast cancer treated with trastuzumab monotherapy and about 50% of patients treated with a combination of trastuzumab with anthracycline and cyclophosphamide exhibit *de novo* resistance [5]. Moreover, although combination of trastuzumab with chemotherapy has significantly improved disease-free survival and overall survival in patients with early-stage HER2+ breast cancer, in the metastatic setting acquired resistance occurs within a year of initial treatment [6]. Treatment of patients with metastatic HER2+ breast cancer with trastuzumab plus lapatinib (EGF104900) provides overall survival advantage over lapatinib monotherapy [7]. Moreover, in the neoadjuvant setting treatment with trastuzumab plus lapatinib (Neo-ALTTO) [8] and trastuzumab plus pertuzumab (Neosphere) [9] results in improved pathological complete response. These data suggest that combination targeted therapies have great potential.

The co-chaperone protein Bcl-2-associated athanogene 1 (BAG-1) exists as three main isoforms BAG-1S, BAG-1M, and BAG-1L and is frequently overexpressed in breast cancer and preinvasive breast disease [10–13]. Clinical studies show that increased BAG-1 immunoreactivity is an independent predictor of outcome particularly in node-positive patients with oestrogen receptor (ER) positive breast cancer receiving adjuvant hormonal therapy alone and enhances the predictive power of IHC4 score (a combination of prognostic information derived from ER, PgR, Ki67, and HER2 immunohistochemical staining) [14–16]. Furthermore, BAG-1 mRNA has been incorporated as a prognostic biomarker in Oncotype DX [17] and PAM50 [18] multigene assays. In breast xenograft studies, BAG-1 overexpression drives growth of oestrogen-responsive ZR-75–1 breast cancer cells in an oestrogen-dependent manner [19]. At a cellular level BAG-1 can promote cancer progression which is characterized by evasion of apoptosis, through the emergence of chemo-resistance [20] and self-sufficiency in growth signals, as shown by growth-factor independent survival [19]. BAG-1 influences cellular function through its interaction with diverse molecular targets including Bcl-2 [21], Hsc70/Hsp70 chaperones [22], ER [14] and RAF-1 [23], a key downstream component of the HER2 signaling pathway.

Although the significance of BAG-1 as a biomarker in ER+ breast cancer is recognized, little is known about the role of BAG-1 in HER2+ disease. BAG-1 protein levels are increased in some HER2+ breast cancer cell lines [10, 24], while HER2 gene transfer in MCF7 cells increases expression levels of BAG-1 and its interacting partner Bcl-2 [25, 26]. Proof-of-principle studies from our laboratory show that it is possible to restrict breast cancer cell growth by targeting BAG-1 protein-protein interactions using synthetic peptides and small molecule compounds, like Thioflavin S (Thio-S) and its biologically potent constituent Thio-2 [27–29]. Our investigation adopted a multipronged strategy comprising overexpression, RNA interference, and protein-protein interaction inhibitors of BAG-1 to examine BAG-1 function in HER2+ breast cancer cells and to explore whether combination of BAG-1-targeted therapies with trastuzumab could restrict growth of these cells more effectively than trastuzumab monotherapy.

## RESULTS

### BAG-1 mRNA and breast cancer outcome

**Figure 1:**
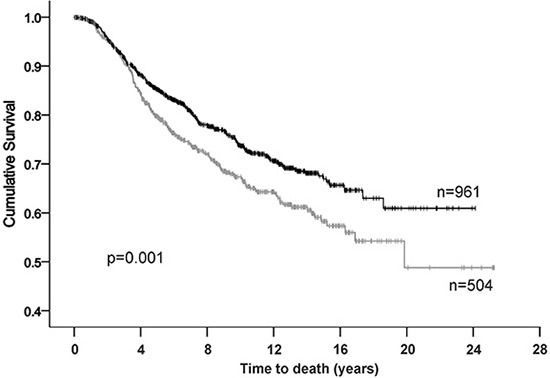
Kaplan-Meier analysis for breast cancer specific survival in the METABRIC cohort High BAG-1-censored (**|**), low BAG-1-censored (**|**).

As expression of BAG-1 protein is frequently increased in breast cancer [12, 14, 15, 30, 31], we examined whether an association might exist between BAG-1 mRNA levels and disease outcome. Oncomine^™^ (Compendia Bioscience, Ann Arbor, MI) was used to analyze BAG-1 gene expression in the Molecular Taxonomy of Breast Cancer International Consortium (METABRIC) dataset [32], comprising 1971 patients of which there were 506 deaths due to breast cancer. An unbiased estimation of the optimal cutpoint between patients whose tumors express BAG-1 at high and at low levels was performed using X-tile software [33]. Statistical significance for death from disease was determined using Kaplan-Meier (log-rank test) univariate analysis (Figure 1). High BAG-1 mRNA expression was significantly associated (*p* = 0.001) with improved prognosis in line with findings from other patient cohorts [15]. Furthermore, high BAG-1 expression remained an independent prognostic predictor (*p* = 0.022) of death from breast cancer in a multivariate model comprising standard clinicopathological variables (Table 1).

**Table 1:** Multivariate analysis of death from breast cancer in the METABRIC cohort comprising standard prognostic markers including BAG-1 mRNA levels

Exploratory variable	Hazard ratio	95% CI of HR	*P* value
BAG-1 status(positive or negative)	0.81	0.67–0.97	0.022
Tumor size(T1, T2, T3)	1.56	1.33–1.84	< 0.001
Lymph node status(positive or negative)	2.14	1.76–2.60	< 0.001
Tumor grade(1, 2, 3)	1.32	1.11–1.56	0.001
ER status(positive or negative)	0.75	0.61–0.93	0.0076
HER2 status(positive or negative)	1.42	1.17–1.74	< 0.001

### BAG-1 expression in HER2+ breast cancer cells

**Figure 2:**
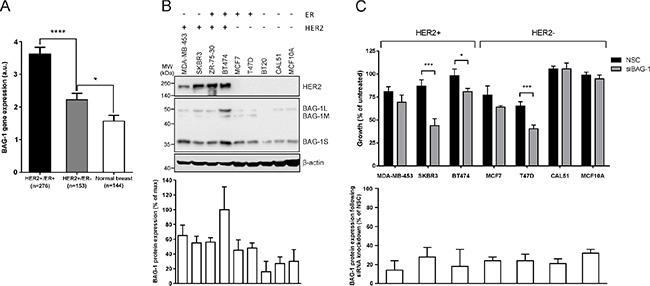
HER2 overexpression upregulates BAG-1 protein expression, while BAG-1 knockdown attenuates growth in HER2+ breast cancer cells (**A**) Bar graph of the METABRIC data set shows the mean ± SEM BAG-1 mRNA expression in HER2+/ER+ and HER2+/ER- breast tumors and in normal breast tissue. One-way ANOVA with Holm-Sidak multiple comparisons test was used for comparison between different groups; *p < 0.05, ****p < 0.0001. (**B**) A representative immunoblot shows endogenous HER2 and BAG-1 expression in a panel of breast cancer cell lines representing different disease subtypes; β-actin was used as a loading control. Densitometric analysis of immunoblots from 3 independent experiments was used to determine expression of total BAG-1 protein which is expressed on the bar graph as a percentage of the maximal value ± SD. (**C**) Effect of nonsense control (NSC) and BAG-1 siRNA (siBAG-1; 50 nM) knockdown on the growth of breast cancer cell lines 6 days post-transfection. Data shown on the top graph are the mean ± SEM from 3 independent experiments, each with three technical replicates. Unpaired *t*-test was used for comparison between NSC and siBAG-1 treatments for each cell line; *p < 0.05, ***p < 0.001. Densitometric analysis of immunoblots from those experiments was used to determine total BAG-1 protein expression following siRNA knockdown and is expressed on the bottom graph as a percent of the corresponding NSC treatment for each cell line ± SD.

Amplification of HER2 is associated with poor prognosis in breast cancer [3]. To examine the association between BAG-1 gene expression and HER2 status the METABRIC dataset was analyzed. Data revealed that BAG-1 mRNA was significantly increased in HER2+/ER+ and HER2+/ER- tumors compared to normal breast tissue (Figure 2A). Survival data for HER2 positive tumours was not analyzed as a separate subgroup as we felt that the number of patients in the HER2 positive subset would not have been sufficient to achieve adequate statistical power for this analysis. We next examined BAG-1 protein immunoreactivity in a panel of breast cancer cell lines representing distinct disease subtypes and in non-tumorigenic MCF10A mammary epithelial cells. Immunoblot analysis showed that BAG-1 protein isoforms were present in all cell lines examined and densitometric analysis revealed that total BAG-1 levels were higher in HER2-overexpressing cells (Figure 2B), in concordance with the METABRIC patient data (Figure 2A). To determine the functional significance of BAG-1 expression in breast cancer, the effect of BAG-1 knockdown by siRNA (siBAG-1) on cell growth was examined in the breast cancer cell line panel. Robust knockdown of BAG-1 protein isoforms was achieved (72%–86%; Figure 2C) and was sustained for at least 8 days post-transfection (data not shown). Following siBAG-1 treatment, growth of HER2-overexpressing MDA-MB-453 was slightly reduced, while that of SKBR3 and BT474 was significantly reduced compared to siRNA nonsense control sequences (NSC) (Figure 2C). Moreover, growth of ER+ T47D cells was significantly reduced, while there was a small but not significant reduction in the growth of MCF7 cells. Triple negative CAL51 and MCF10A non-tumorigenic cells remained unaffected (Figure 2C). Under these conditions, growth of ZR-75–30 and BT20 cells was adversely affected by NSC control thereby precluding meaningful interpretation. Taken together, these data suggest that BAG-1 may have a prosurvival role in HER2+ cells and further justifies investigating the impact of BAG-1 on the sensitivity of HER2+ cells to trastuzumab and the potential of BAG-1 as a therapeutic target in HER2+ breast cancer.

### Effect of BAG-1 overexpression and knockdown on the growth of HER2+ cells treated with trastuzumab

**Figure 3:**
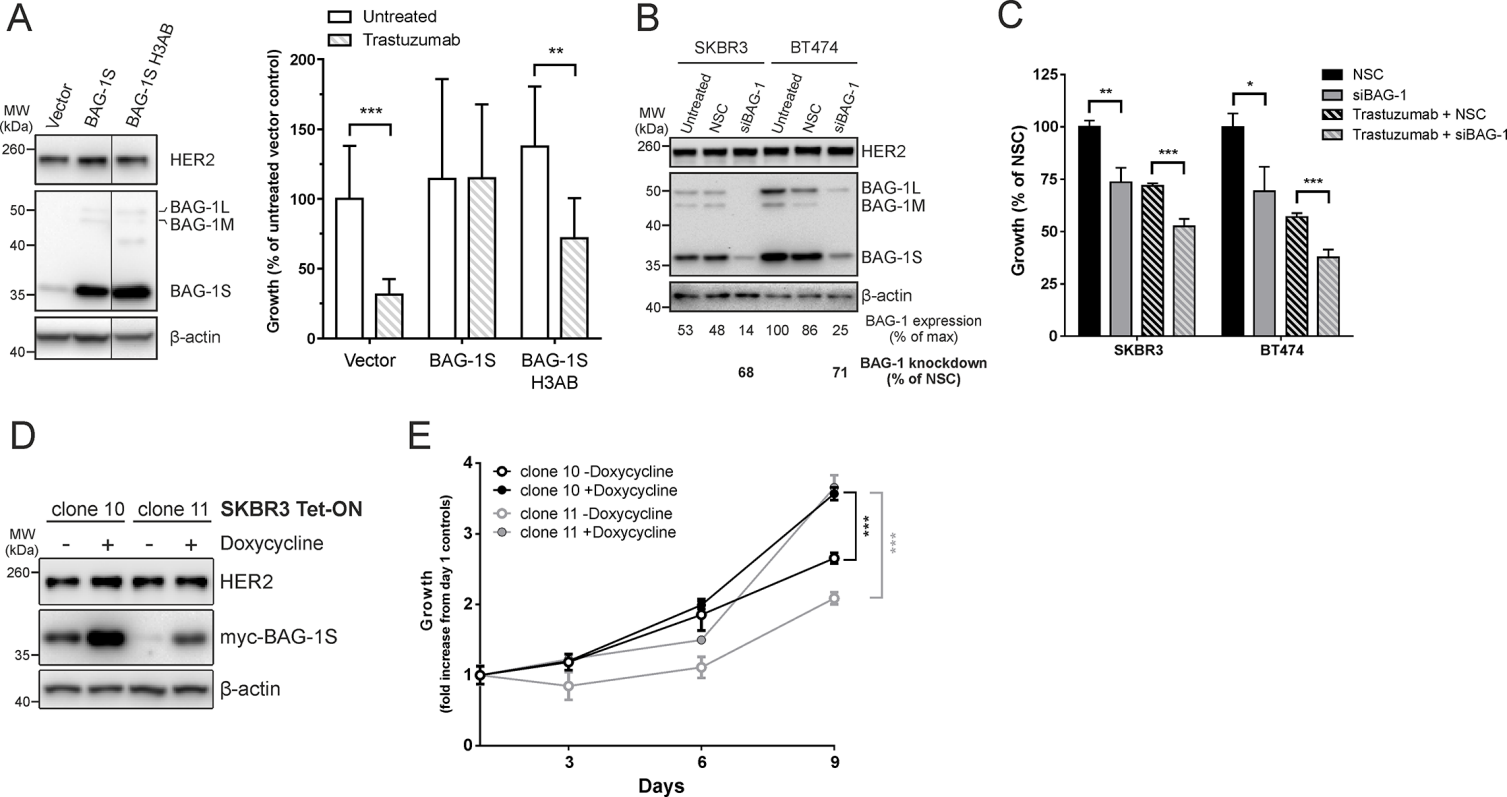
BAG-1 overexpression attenuates the growth inhibitory effect of trastuzumab while BAG-1 knockdown potentiates the effect of trastuzumab (**A**) A representative immunoblot shows BAG-1 and HER2 protein expression in SKBR3 cells 96 h post-transfection with the indicated constructs; β-actin was used as a loading control. Bar graph shows the effect of trastuzumab (8 μg/ml) on the long-term growth potential of SKBR3 cells transfected with vector control (pcDNA3) or pcDNA3 containing BAG-1S or BAG-1S H3AB (BAG domain mutant incapable of interacting with HSC70). Transfectants were maintained in complete media supplemented with G418 for 2 weeks. Data on the bar graph show the mean ± SD and are representative of two independent experiments, each with three technical replicates. Unpaired *t*-test was used for comparison between different treatments; **p < 0.01, ***p < 0.001. (**B**) A representative immunoblot shows BAG-1 and HER2 expression levels in SKBR3 and BT474 cells 3 days following siRNA (50 nM) knockdown; β-actin was used as a loading control. Densitometric analysis of immunoblots from 3 independent experiments was used to determine expression of total BAG-1 protein and is expressed as a percentage of the maximal value. The amount of BAG-1 protein knockdown by siBAG-1 is expressed as a percentage of the corresponding NSC treatment for each cell line. (**C**) SKBR3 and BT474 cells transfected with NSC or siBAG-1 were grown for 5 days with or without trastuzumab (32 μg/ml) and cell growth was measured using crystal violet assay. Data are the mean ± SEM from 4 independent experiments, each with three technical replicates. Unpaired *t*-test was used for comparison between different treatments; *p < 0.05, **p < 0.01, ***p < 0.001. (**D**) A representative immunoblot shows induction of Tet-On myc-BAG-1S using an anti-myc antibody in SKBR3 cell clones 10 and 11 48 h after addition of doxycycline (2 μg/ml); β-actin was used as a loading control. (**E**) Line graph shows the effect of BAG-1S induction by doxycycline (2 μg/ml) on cell growth over 9 days using crystal violet assay. Data shown on the graph are the mean ± SEM from 3 independent experiments, each with three technical replicates. Unpaired *t*-test was used for comparison between doxycycline induced and uninduced cells; ***p < 0.001.

To investigate the possibility that targeting BAG-1 protein function in HER2+ cells could influence response to trastuzumab, BAG-1S, which has previously been shown to protect MCF7 cells from a variety of stresses [20], was overexpressed in SKBR3 HER2+ cells as verified by immunoblot analysis (Figure 3A); an empty vector was transfected as a control (Figure 3A). HER2 expression levels remained unchanged 96 h post-transfection in control and BAG-1S transfected cells (Figure 3A). Although treatment with trastuzumab resulted in 70% reduction in the long-term growth of control cells (Figure 3A) this was inhibited by overexpression of BAG-1S, as reflected by the similar level of growth observed between untreated and trastuzumab-treated BAG-1S overexpressing cells (Figure 3A). These data suggest that BAG-1S overexpression can inhibit the growth-inhibitory effect of trastuzumab.

We also examined whether response to trastuzumab could be influenced by BAG-1 protein interactions. To this end, a BAG domain helix 3 mutant (Q201A/D208A/Q212A) BAG-1S protein (BAG-1S H3AB), which is defective in binding HSC70 [34], was overexpressed in SKBR3 cells as confirmed by immunoblot analysis (Figure 3A). Studies using a series of BAG-1 deletion mutants have shown that although helix 3 of the BAG domain, the region within which the H3AB mutation occurs, mediates interaction with HSP70 it does not appear to be important for interaction with RAF-1 [23]. Overexpression of BAG-1S H3AB did not affect HER2 protein expression but resulted in 50% growth reduction in response to trastuzumab compared to untreated cells (Figure 3A). These data suggest that growth inhibition by trastuzumab relies in part on the interaction of BAG-1S with HSC70 protein chaperones.

We reasoned that a reduction of BAG-1 protein expression would potentiate the growth-inhibitory effect of trastuzumab. SKBR3 and BT474 cells, which have high endogenous BAG-1 expression (Figure 2B) and whose growth is susceptible to targeting with siBAG-1 (Figure 2C), were selected to address this. Immunoblot analysis revealed that siBAG-1 resulted in a marked decrease in BAG-1 expression levels compared to NSC but had no effect on HER2 expression (Figure 3B). In addition, BAG-1 knockdown resulted in ∼25% decrease in the growth of both SKBR3 and BT474 cells relative to NSC (Figure 3C). When combined with trastuzumab, siBAG-1 caused a further ∼20% reduction in growth in both cell lines compared to trastuzumab+NSC control (Figure 3C).

As transient transfections can lead to heterogeneous and unsustained protein expression, we generated a SKBR3 Tet-On myc-BAG-1S inducible cell line in order to further corroborate the protective role of BAG-1 in response to trastuzumab; a myc tag was chosen in order to differentiate overexpressed from endogenous BAG-1S. Two clones were generated which fortuitously exhibited differential basal (−Doxycycline) levels (Figure 3D) enabling us to examine the effect of basally overexpressed BAG-1S on cell growth. Addition of doxycycline resulted in upregulation of myc-BAG-1S protein expression but did not affect HER2 protein levels (Figure 3D). Growth rate correlated with the level of BAG-1S overexpression in both induced and uninduced conditions. In the absence of doxycycline, clone 10 grew faster than clone 11 (Figure 3E). Upon induction with doxycycline clone 11 grew faster, relative to its corresponding uninduced control, versus clone 10 which exhibited higher BAG-1S basal expression levels (Figure 3D and 3E). Increased expression of BAG-1S therefore results in significantly increased SKBR3 growth rate.

### Effect of BAG-1 overexpression on the growth of HER2+ mammary xenografts

**Figure 4:**
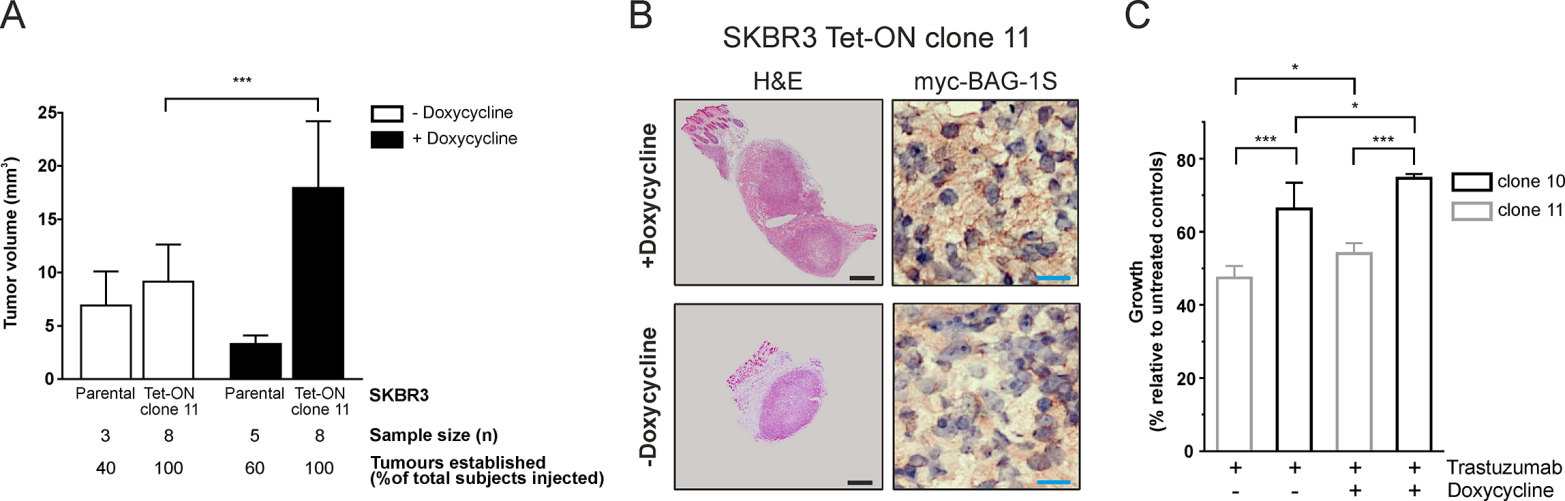
Inducible expression of BAG-1S promotes growth of SKBR3 HER2+ tumors in the mouse breast (**A**) SKBR3 parental and Tet-On myc-BAG-1S clone 11 cells were injected as a PBS:Matrigel (1:1) suspension into the mammary fat pads of NOD. SCID mice, which were given doxycycline-containing (+Doxycycline; 200 μg/ml) or plain water (−Doxycycline, control) *ad libitum*, and were allowed to form tumor xenografts over 32 days. Data shown on the bar graph are the mean ± SD tumor volume from 3 independent experiments. Unpaired *t*-test was used for comparison between different groups; ***p < 0.001. (**B**) Representative examples of SKBR3 Tet-On myc-BAG-1S xenografts to illustrate haematoxylin and eosin (H & E) tumor staining and immunohistochemical staining for myc-BAG-1S in the presence (+Doxycycline) or absence (−Doxycycline) of inducer following 32 days of growth in the mammary fat pads of mice. Black and blue size bars represent 500 μm and 20 μm respectively. (**C**) Effect of trastuzumab (32 μg/ml) on the growth of doxycycline-induced (+Doxycycline; 2 μg/ml) or uninduced (−Doxycycline) Tet-On SKBR3 myc-BAG-1S clones after 5 days of treatment using crystal violet assay. Data shown are the mean ± SEM of a representative experiment with three technical replicates; similar results were obtained in a second experiment. One-way ANOVA with Holm-Sidak multiple comparisons test was used for comparison between different groups; *p < 0.05, ***p < 0.001.

To investigate the role of BAG-1 in HER2+ tumorigenesis in the breast, Tet-On myc-BAG-1S inducible clone 11 or parental SKBR3 cells were injected into the mammary fat pads of mice that received doxycycline-supplemented (induced) or normal water (uninduced) and were allowed to form tumors over 32 days. Induced Tet-On myc-BAG-1S tumors were 2-fold larger (mean ± SD: 18.0 ± 6.2 mm^3^ vs 9.1 ± 3.5 mm^3^) (Figure 4A) than uninduced controls. A comparison between doxycycline-treated Tet-On myc-BAG-1S and parental SKBR3 tumors, to account for the net effect of the inducer on the cells, revealed a 5.5-fold increase in size (mean ± SD: 3.3 ± 0.8 mg vs 18 ± 6.2 mg) (Figure 4A). Haematoxylin and eosin staining revealed that both induced and uninduced tumors exhibited poorly differentiated architecture (Figure 4B). Immunoreactivity of myc-BAG-1S appeared granular, localised to the cytoplasm, and was overall greater in the induced setting (Figure 4B). Staining for pancytokeratin, Ki67, and platelet endothelial cell adhesion molecule 1, a marker indicating mouse vasculature formation within the tumor, was present at a similar intensity in the area around the centre of all tumors examined (data not shown). These data suggest that increased expression of BAG-1S in HER2+ SKBR3 cells promotes tumor growth *in vivo*.

We next examined the effect of trastuzumab on the growth of SKBR3 cells *in vitro*. When treated with trastuzumab, growth of doxycycline-induced clones 10 and 11, which overexpress myc-BAG-1S (See Figure 3D), was greater than that of uninduced controls and correlated with the level of BAG-1S overexpression, as clone 10 exhibited overall significantly more growth than clone 11 cells (Figure 4C). These data suggest that induction of BAG-1S overexpression attenuates growth inhibition by trastuzumab *in vitro*.

### Effect of combining BAG-1-targeting therapies with trastuzumab on HER2+ cell growth

**Figure 5:**
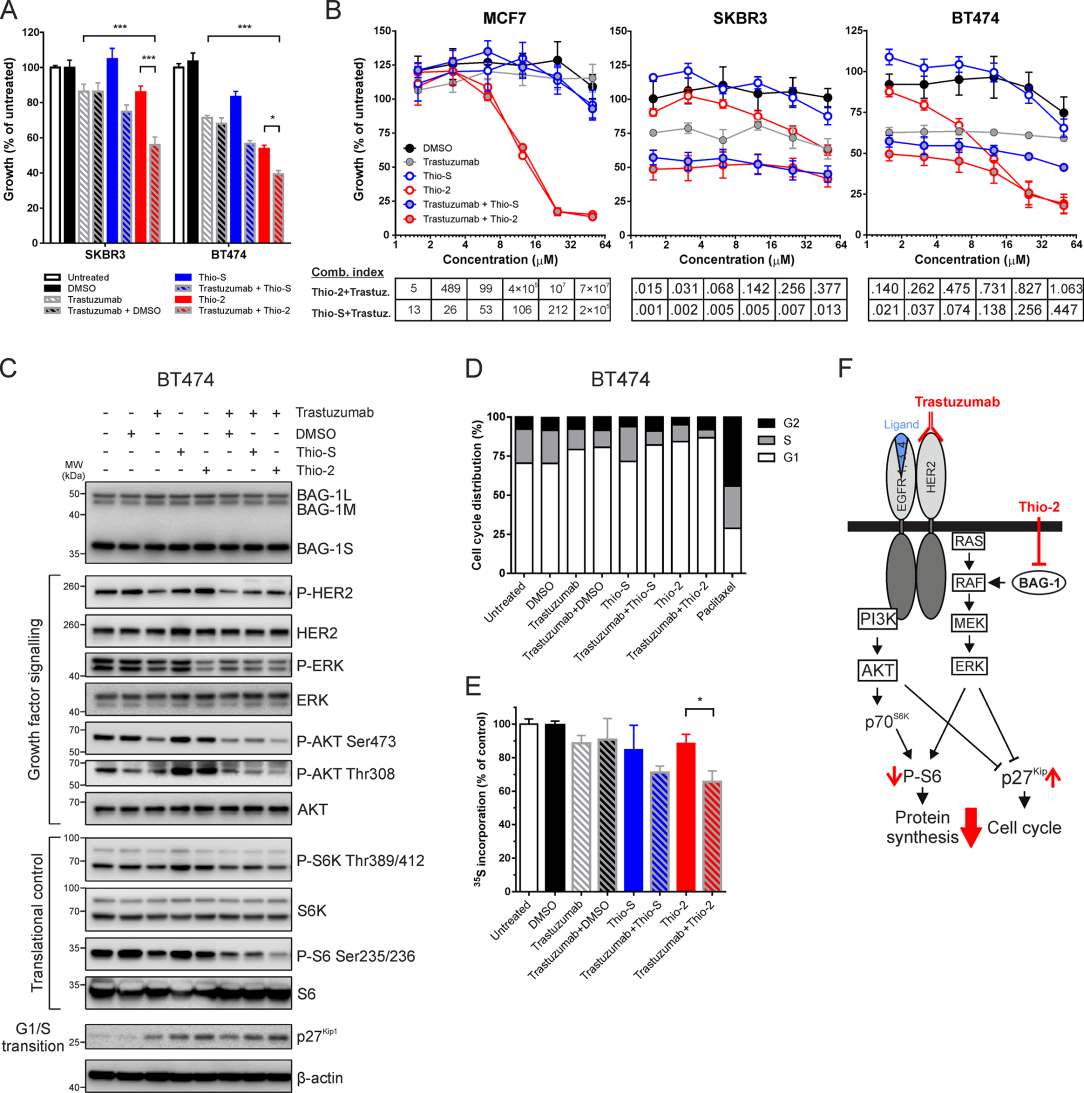
Synergistic effect of targeting BAG-1 and HER2 on breast cancer cell growth and signaling (**A**) SKBR3 and BT474 cells were treated for 5 days with DMSO (0.125% v/v), trastuzumab (16 μg/ml), Thio-S (12.5 μM), Thio-2 (12.5 μM) or combinations of these compounds, as indicated. Cell growth was measured using crystal violet assay and is expressed as a percentage of untreated cells. Data shown on the graph are the mean ± SEM from 3 independent experiments, each with three technical replicates. Statistical significance was determined using two-way ANOVA with Holm-Sidak multiple comparisons test. *p < 0.05, ***p < 0.001. (**B**) MCF7, SKBR3 and BT474 cells were treated over 5 days with increasing concentrations of Thio-S, Thio-2, and/or trastuzumab at a constant ratio. Cell growth was measured by crystal violet assay and is expressed as a percentage mean ± SEM of untreated cells. Combination indexes were determined by CalcuSyn v2.11; values < 1 indicate synergism. (**C**) A representative immunoblot of 3 independent experiments shows the effect of single compounds and compound combinations (concentrations listed in Figure 5A), 24 h after treatment, on the phosphorylation and expression levels of molecules involved in growth factor signaling and translation/cell cycle control downstream of HER2 and BAG-1; β-actin was used as a loading control. (**D**) BT474 cells were treated with single compounds or compound combinations (concentrations listed in Figure 5A) for 48 h and were subsequently harvested and stained with propidium iodide for cell cycle analysis (20,000 events/sample) using ModFit LT v4.1.7. Bar graph shows the proportion of cells in each phase of the cell cycle as a percentage of the total number of cells stained. Paclitaxel (100 nM) treatment for 24 h was used as a positive control for cell cycle arrest. Data are representative of three independent experiments. (**E**) BT474 cells were metabolically labelled with ^35^S to quantify global protein synthesis following 24 h treatment with the compounds indicated (concentrations listed in Figure 5A). ^35^S incorporation corresponding to the total amount of protein synthesis is expressed as a percentage of diluent control. Data shown on graph are the mean ± SEM from 3 independent experiments, each with three technical replicates. Unpaired *t*-test was used for comparison between different treatments; *p < 0.05. (**F**) Simplified schematic diagram of PI3K/AKT and RAF/MEK/ERK signaling pathways downstream of HER2 and BAG-1. Red arrows indicate the molecular changes which occur as a result of combining trastuzumab with Thio-2, an inhibitor of BAG-1 protein-protein interactions.

As BAG-1 knockdown by siRNA enhanced the growth-inhibitory effect of trastuzumab, we examined whether using Thio-S (a mixture of compounds) and its biologically potent constituent Thio-2, which have been shown to inhibit BAG-1 protein-protein interactions [27, 29], would have a similar effect. To reduce any potential non-specific effects of the compounds targeting BAG-1 and to keep the concentration of compound diluent (DMSO) in culture to a minimum, we used a 4-fold lower concentration than previously reported [27, 29] based on compound titrations we performed. Treatment of SKBR3 or BT474 cells with trastuzumab and Thio-2 combination resulted in significantly reduced growth compared to single compound treatments (Figure 5A); the effect of trastuzumab+Thio-2 was overall more pronounced compared to trastuzumab+Thio-S. Treatment of SKBR3 or BT474 with a combination of Rituximab (a humanised anti-CD20 antibody used here as control for antibody-dependent cellular cytotoxicity) with Thio-2 or Thio-S, had no additional effect on growth than either of those BAG-1 inhibitors used in isolation (data not shown).

To examine the enhanced growth inhibition observed in response to BAG-1 and HER2 targeted therapies, cells were treated with increasing concentrations of single compounds or compound combinations at a constant ratio. Statistical analysis of the efficacy of compound interactions revealed a synergistic mode of action for both trastuzumab+Thio-S and trastuzumab+Thio-2 combinations, in BT474 and SKBR3 HER2+ but not in MCF7 HER2- cells. In particular, BT474 were more susceptible than SKBR3 in response to Thio-2 treatments (Figure 5B). To elucidate the nature of this synergy at the protein signaling level, immunoblot analysis was performed to examine the molecular changes that occur when targeting BAG-1 and HER2 in BT474 cells; compound treatments were applied for 24 h, similarly to studies by other groups [35]. Treatment with trastuzumab alone resulted in a decrease in the phosphorylation of HER2, AKT^Ser473^, ERK, p70 S6 kinase (S6K), and ribosomal S6 protein (Figure 5C). Moreover, phosphorylation of ERK and S6 was noticeably reduced by Thio-2 treatment alone and no clear difference with Thio-S alone was observed. Additionally, phosphorylation of AKT^Ser473^ was slightly reduced by Thio-2 (Figure 5C). Single treatment with either Thio-2 or Thio-S caused a marked upregulation of Akt^Thr308^ and S6K phosphorylation compared to DMSO control, while trastuzumab+Thio-2 combination treatment caused a reduction in the phosphorylation of AKT^Ser473/Thr308^, ERK, S6K, and S6 compared to trastuzumab+DMSO control (Figure 5C). In comparison, trastuzumab+Thio-S caused a smaller reduction in the phosphorylation of AKT^Thr308^, ERK, and S6 but had no effect on AKT^Ser473^ compared to trastuzumab+DMSO control. Furthermore, as shown on the immunoblot in Figure 5C, cell treatment with single compounds or compound combinations led to a noticeable increase in the expression levels of p27^Kip1^, an inhibitor of G1/S cell cycle transition [36], which was greatest in the presence of Thio-2.

Analysis of cell cycle using propidium iodide staining was therefore performed to elucidate the growth-inhibitory effect observed in response to combination treatment targeting BAG-1 and HER2. Results show that trastuzumab caused G1/S arrest in BT474 cells, while Thio-2 treatment exhibited greater growth arrest (Figure 5D). A further increase in G1/S arrest was observed when trastuzumab and Thio-2 were used in combination. Thio-S did not induce G1/S arrest on its own but caused slightly more G1/S arrest when combined with trastuzumab compared to control (trastuzumab+DMSO) (Figure 5D). There was no sub-G1 cell fraction, an indicator of cell death, detected in any of the examined conditions (data not shown).

S6 protein is an indispensable component of the 40S ribosomal subunit, and is thought to be involved in regulating translation [37]. Based on the changes we observed on S6 phosphorylation in response to treatment, we further investigated the effect of targeting HER2 and/or BAG-1 on global protein biosynthesis by ^35^S metabolic labelling of BT474 cells. Treatment with trastuzumab, Thio-2, or Thio-S reduced protein synthesis compared to controls (Figure 5E). When compared to single compound treatments trastuzumab+Thio-2 combination significantly attenuated translation, while trastuzumab+Thio-S treatment had a similar but less pronounced effect (Figure 5E). Overall, these data show that combining trastuzumab with the BAG-1 protein-protein interaction inhibitor Thio-2 synergistically reduces growth of BT474 cells through suppression of ERK and AKT signaling pathways and leads to a reduction in global protein synthesis and G1/S arrest (Figure 5F).

### Effect of BAG-1 inhibition on trastuzumab-resistant cells

**Figure 6:**
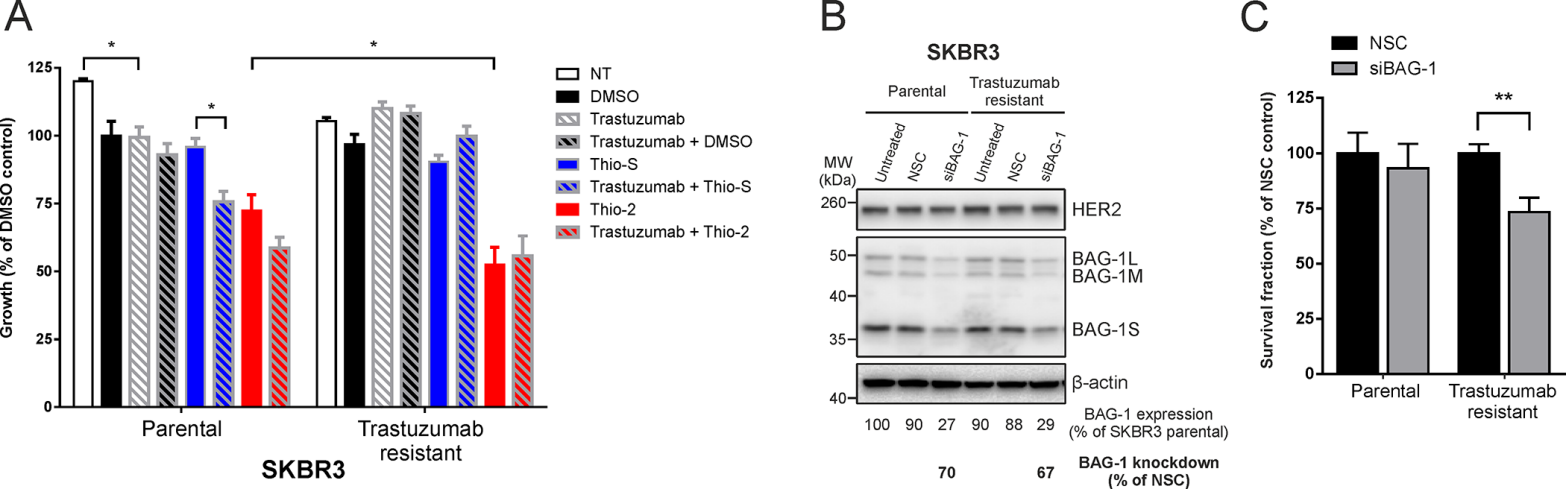
Trastuzumab-resistant SKBR3 cells are sensitive to BAG-1 inhibition (**A**) SKBR3 parental or trastuzumab-resistant cells were treated over 5 days with DMSO (0.5% v/v), trastuzumab (32 μg/ml), Thio-S (50 μM), Thio-2 (50 μM), or combinations of these compounds as indicated. Cell viability was measured using crystal violet assay and is expressed as a percentage of DMSO control. Data shown on the graph are the mean ± SEM from 3 independent experiments, each with three technical replicates. Statistical significance was determined using two-way ANOVA with Holm-Sidak multiple comparisons test. *p < 0.05. (**B**) A representative immunoblot shows BAG-1 and HER2 expression levels in SKBR3 parental and trastuzumab resistant cells 4 days following siRNA (50 nM) knockdown. Densitometric analysis of immunoblots from 3 independent experiments was used to determine expression of total BAG-1 protein and is expressed as a percentage of untreated SKBR3 parental cells. The amount of BAG-1 protein knockdown by siBAG-1 is expressed as a percentage of the corresponding NSC treatment for each cell line; β-actin was used as a control for loading. (**C**) Bar graph shows the effect of BAG-1 knockdown by siRNA (50 nM) on the long-term growth potential of SKBR3 parental or trastuzumab-resistant cells. Data shown on graphs are the mean ± SEM from 3 independent experiments, each with 3 technical replicates. Unpaired *t*-test was used for comparison between different treatments; **p < 0.01.

We next addressed whether BAG-1 targeting might be of benefit in cases where acquired resistance to trastuzumab has developed. To this end, we examined the effect of targeted BAG-1 therapies on the growth of trastuzumab-resistant SKBR3 cells characterized by Nahta *et al.* [35] in which insulin growth factor receptor 1, a protein associated with resistance to trastuzumab [38], interacts with and modulates the activity of HER2. Parental cells responded to trastuzumab by a ∼20% reduction in growth compared to untreated control (Figure 6A); a further reduction in growth was observed in response to trastuzumab+Thio2 and trastuzumab+Thio-S combination therapies compared to single treatments with trastuzumab, Thio-2, or Thio-S (Figure 6A). As expected, trastuzumab-resistant cells were unresponsive to trastuzumab but responded significantly more to Thio-2 than parental cells (Figure 6A); combination treatment (trastuzumab+Thio-2) did not reinstate sensitivity to trastuzumab.

To corroborate these findings and examine the role of BAG-1 in the long-term growth potential of trastuzumab-resistant cells, a siRNA knockdown approach was used. Following siRNA transfection, viable parental or trastuzumab-resistant SKBR3 cells were allowed to grow over 2 weeks. Immunoblot analysis revealed approximately 70% knockdown in BAG-1 protein levels (Figure 6B). Quantification of long-term growth showed that trastuzumab-resistant cells were more susceptible to treatment with siBAG-1, as indicated by a reduction in their overall growth, compared to similarly treated parental cells (Figure 6C). Altogether, our data suggest that BAG-1 can partially support growth of these trastuzumab-resistant cells. Targeting BAG-1 might therefore constitute a potential therapeutic option for HER2+ breast cancers refractory to trastuzumab.

## DISCUSSION

In this study we show for the first time that elevated BAG-1 protein expression correlates with that of HER2, is important for optimal growth of some HER2-overexpressing breast cancer cells, and impedes the growth-inhibitory effect of trastuzumab. Moreover, inducible overexpression of BAG-1S results in the growth of larger poorly differentiated HER2+ tumor xenografts in the mammary fat pads of mice, while induction of BAG-1S expression in *in vitro* culture attenuates growth inhibition by trastuzumab. Combination treatment targeting BAG-1 with small molecule protein-protein interaction inhibitors and HER2 with trastuzumab synergistically reduces breast cancer cell growth, which is accompanied by attenuation of protein synthesis and induction of G1/S cell cycle arrest through the ERK and AKT pathways.

Analysis of the METABRIC patient cohort [32] revealed that BAG-1 mRNA is increased in HER2+/ER+ as well as HER2+/ER- breast tumors compared to normal breast epithelium, and in HER2+ cell lines BAG-1 protein expression is elevated. The observed increase in BAG-1 expression in HER2+ breast cancer cells raised the possibility that certain HER2+ cells may be dependent on BAG-1 for growth in response to the HER2-targeted therapy trastuzumab. Knocking down BAG-1 expression by siRNA confirmed that SKBR3 and BT474 HER2+ cell lines require BAG-1 for optimal growth. Based on this finding and the fact that induction of BAG-1 expression leads to the formation of larger xenografts in the mammary fat pads of mice, we hypothesized that these tumors may be more resistant to trastuzumab therapy depending on the level of BAG-1 expression. In support of this, we present evidence from *in vitro* culture experiments showing that induction of BAG-1S expression in two SKBR3 cell clones leads to an increase in their growth rate and impedes growth inhibition by trastuzumab in a manner that correlates with the level of BAG-1S overexpression. Moreover, overexpression of BAG-1S offered protection in long-term SKBR3 cell cultures after transient exposure to trastuzumab, suggesting that upregulation of BAG-1 may be a way by which cells could develop resistance to this drug. This protection is partly due to the interaction between BAG-1S and HSC70/HSP70 as a BAG domain helix 3 mutant (BAG-1S H3AB), which is deficient in chaperone binding, is sensitive to treatment with trastuzumab. These findings are in agreement with data published previously by our group showing that BAG-1S overexpression prevents heat shock-induced long-term growth inhibition in MCF7 cells through its interaction with HSC70/HSP70 protein chaperones [20]. Our proof-of-principle data show that attenuating endogenous BAG-1 expression by siRNA in HER2+ cells which are partly dependent on it for growth further increases the growth-inhibitory effect of trastuzumab. Our data indicate that BAG-1 overexpression can support growth of HER2+ breast cancer cells and reduces the growth-inhibitory effect of trastuzumab both in short and long-term cultures. In contrast, targeting endogenous BAG-1 function with siRNA impedes growth of these cells and potentiates the growth-inhibitory effect of trastuzumab. These results provide a rationale for assessing BAG-1 as a biomarker of therapeutic response in clinical studies of breast cancers treated with trastuzumab.

Based on our current data and previous findings [20, 27, 29] from our group we hypothesised that using Thio-S or Thio-2 inhibitors of BAG-1 protein-protein interactions in combination with trastuzumab, a first-line treatment for HER2+ breast cancer [4], would further restrict growth of HER2+ breast cancer cells. The synergistic growth-inhibitory effect observed at the dose used suggested that the prosurvival function of BAG-1 and HER2 may be exerted on distinct but functionally convergent signaling pathways. BAG-1 is known to activate RAF [23] and we have previously reported [29] that Thio-2 acts at the level of RAF to disrupt BAG-1 protein-protein interactions (Figure 5F). Here, we found that single Thio-2 treatment markedly inhibited ERK but had comparatively little effect on AKT activity leading to attenuation of HER2+ breast cancer cell growth. Phosphorylation of ERK was slightly reduced with trastuzumab treatment. Studies have shown that allosteric inhibition of ERK activity exerts a minimal effect on the proliferation of trastuzumab-sensitive SKBR3 and BT474 cells [39] and suggest that the growth-inhibitory effect observed is predominantly driven through the PI3K/AKT signaling pathway leading to a decrease in S6^Ser235/236^ phosphorylation and induction of cell cycle arrest. BAG-1 targeting caused compensatory activation of AKT by phosphorylation at Thr308, which is known to be regulated by PDK1 [40], and resulted in increased S6K activity, which is crucial for protein synthesis and cell cycle progression [41]. As there was little change on AKT^Ser473^ phosphorylation under these conditions, it is less likely that the observed AKT^Thr308^ phosphorylation was caused by mTOR, a key activator of AKT [42]. It is conceivable that this may have been caused by increased activation of HER2, since combination of BAG-1 inhibitors with trastuzumab resulted in a reduction of AKT^Thr308^ and S6K^Thr389/412^ phosphorylation to basal levels. Our data are consistent with other studies showing that when used as single agents for cancer therapy, MEK inhibitors frequently lead to increased AKT phosphorylation by relieving a negative feedback loop of ERK on HER-family receptor signaling, which can result in reduced efficacy [43]. Moreover, trastuzumab caused G1/S cell cycle arrest that was augmented in the presence of Thio-2 but not Thio-S. This is in line with our previous findings [29] showing increased potency of Thio-2 over Thio-S at inhibiting the interactions between BAG-1 with HSC70 and BAG-1 with RAF. This ability of Thio-2 may account for the increased growth inhibition of HER2+ cells.

Our investigation reveals a protective role for BAG-1 in HER2+ breast cancer and provides insight into the mechanism by which increased BAG-1 protein expression supports cell viability by attenuating the growth-inhibitory effect of trastuzumab. Combining BAG-1- and HER2-targeted therapies synergistically inhibits HER2+ breast cancer cell growth, while interfering with BAG-1 function targets trastuzumab-resistant cells more effectively. The concept of targeting BAG-1, a protein that supports cancer cell survival but is also related to improved patient survival, may on the surface seem paradoxical. Nevertheless, these observations are not mutually exclusive; since BAG-1 enhances ER transcription and can stimulate ER activity [14], it would be expected to support growth of breast cancers that are reliant on BAG-1 related pathways. This is similar to the ER in ER+ breast cancer where its expression is associated with good prognosis [44], whilst the ER remains an excellent target for hormonal therapy, for example with tamoxifen. A few clinical implications can be drawn from this study. BAG-1 could be a candidate predictive biomarker by enabling the definition of breast cancer patient subgroups likely to display *de novo* resistance to trastuzumab. Moreover, our data indicate that disrupting BAG-1 function might be a useful therapeutic strategy for targeting HER2+ breast cancers in combination with trastuzumab, but also for those cancers that are refractory to trastuzumab, and emphasise the need for developing drug-like BAG-1 inhibitors and/or testing the efficacy of existing inhibitors of BAG-1 mediated pathways.

## MATERIALS AND METHODS

### Cell culture and treatments

All cell lines were purchased from LGC Standards (UK), while CAL51 were purchased from DSMZ (Germany); cell lines were grown as recommended by their suppliers. SKBR3 Tet-On myc-BAG-1S inducible clones were maintained in DMEM containing 10% (v/v) tetracycline-free FCS (Biosera, France), 2 mM L-Glutamine, penicillin (100 units)/streptomycin (100 μg/ml), and blasticidine/zeocin for selection. Expression of myc-BAG-1S was induced by doxycycline (2 μg/ml). MCF10A cells were cultured as described by Debnath *et al.* [45]. SKBR3 parental and trastuzumab-resistant (clone 3) cells were kindly provided by Professor Francisco Esteva (M. D. Anderson Cancer Center, USA) and cultured as previously described [35]. All cells were maintained at 37°C in a 10% CO_2_ humidified atmosphere. Thioflavin S (Thio-S) practical grade was from Sigma (UK) and Thio-2 was produced at the Institute of Pharmacy/Pharmacognosy, University of Innsbruck as described previously [29]. Trastuzumab was gifted by the Oncology Pharmacy at Southampton General Hospital.

### Expression constructs

pcDNA3-BAG-1S wt and H3AB mutant constructs were generated as described previously by our group [14, 34]. An inducible Tet-On myc-BAG-1S construct was generated by touchdown PCR amplification [46] using primers: 5′- ATTAAGCTTGCCA CCATGGAACAGAA ACTGATCTCTGAAG AAGACCTGAATCGGAGCCAG GAGGTGACCC and 5′- CCGCTCGAGCTGCTACACCT CACTCGG CCAG and subsequent ligation of the resulting amplicon into a pcDNA4/TO plasmid (T-REx System, Invitrogen, UK) with HindIII and XhoI flanking sites.

### Cell transfection

Expression constructs were transfected using Fugene HD (Promega, UK) according to the manufacturer's instructions. ON-TARGETplus SMARTpool BAG-1 siRNA or siRNA nonsense control sequences (GE Healthcare Dharmacon, UK) were transfected by DharmaFECT 1 reagent according to the manufacturer's protocol. Cells were seeded for experiments 24 h post-transfection. To generate Tet-On inducible clones, pcDNA4/TO-myc-BAG-1S:pcDNA6/TR (T-REx^™^ System, Life Technologies, UK) vectors were co-transfected into SKBR3 cells and individual clones were isolated following blasticidine/zeocin selection.

### Cell growth assays

The effect of drugs on cell survival was determined by crystal violet assay. Briefly, 1,000–10,000 cells/well were seeded in triplicate in 96 well plates (Corning, USA) for short-term (4–5 days) cell viability or in 12 well plates for long-term growth assays, as described by Townsend *et al.* [20], and treated as described in the text or figure legends. Uninduced or doxycycline-induced cells were allowed to grow for a further 24 h prior to treatment. Following treatment, media were removed and monolayers fixed in 4% paraformaldehyde (pH 7.4) for 30 min and stained with 0.5% crystal violet for 20 min. Plates were rinsed in a water bath and dried. Growth was expressed as a measure of the adherent cells by extracting the associated dye with 10% (v/v) acetic acid and reading the absorbance at 590 nm with a Varioskan Flash multimode reader (Thermo Scientific, UK). Long-term growth was measured using ColonyArea ImageJ (1.48v) plugin [47] and the fraction of growing cells was expressed as a percent of untreated control as described by Franken *et al.* [48].

### Cell lysis and immunoblotting

Cells were lysed on ice for 30 min in RIPA buffer (CST, UK) containing protease and phosphatase inhibitor cocktails (Sigma, UK). Lysates were clarified by centrifugation (13,000 rpm, 15 min, 4°C), diluted with Laemmli buffer, and denatured at 95°C for 5 min. SDS-PAGE and immunoblotting were performed according to standard protocols [49]. Most primary antibodies used were from CST, BAG-1 (3.10 G3E) was from Santa Cruz Biotechnology, and β-actin-HRP was from Sigma (UK). HRP-conjugated secondary antibodies were from Dako (Denmark). Proteins were detected by SuperSignal West Pico or Fempto chemiluminescent substrate (Thermo Scientific, UK) using a BioRad Fluor-S Multiimager with Quantity One 1-D v4.6.6 image acquisition and analysis software.

### FACS analysis

For cell cycle analysis, cells were processed as described before [35]. DNA content was determined by capturing 20,000 events using a FACSCalibur (Becton Dickinson) flow cytometer following the manufacturer's instructions. The proportion of cells in different phases of the cell cycle was analyzed with ModFit LT v4.1.7 and expressed as a percentage of total cells.

### Metabolic labelling

Tran^35^S-Label (MP Biomedicals, Illkirch, France) was added to the culture medium (0.37 MBq/ml) for the final 2 h of culture. Cells were lysed in RIPA buffer with protease inhibitors. Lysates were supplemented with 10 mg/ml L-cysteine and 10 mg/ml L-methionine, clarified by centrifugation, applied onto Whatmann filter discs (GE Healthcare, UK), and air dried. Bound proteins were precipitated by 10% (w/v) trichloroacetic acid (Sigma, UK). Boiling 5% (w/v) trichloroacetic acid was added to the filter discs before washing in absolute ethanol and acetone. The filter discs were air-dried, immersed in OptiScint ‘HiSafe’ scintillation fluid (PerkinElmer, UK) and radioactivity was measured using a WALLAC 1409 liquid scintillation counter (PerkinElmer, USA). Control cells were treated with cycloheximide (10 μg/ml). Counts from cycloheximide-treated samples were subtracted from experimental values and the difference was expressed as a percentage of untreated cells.

### Tumor xenografts and immunohistochemistry

NOD.SCID mice (Harlan Laboratories, UK) were bred and maintained locally under pathogen-free conditions. Animal experiments were approved by the local ethical committee and performed under Home Office license PPL30/2964. Two days before tumor cell injection, female animals were offered *ad libitum* doxycycline-containing (200 μg/ml) water for induction of myc-BAG-1S expression or plain water as control. For tumor implantation, SKBR3 Tet-On myc-BAG-1S cells were mixed 1:1 with BD Matrigel basement membrane matrix (SLS, UK). Cell suspensions (5 × 10^6^ cells/mouse) were injected subcutaneously into the mammary fat pad and allowed to form xenografts over 32 days. Tumors were measured by callipers, weighed, and cryopreserved in OCT buffer (CellPath, UK). Haemmatoxylin and eosin (H & E) staining was performed as previously described [50] and immunohistochemical detection was performed using ImmPress HRP polymer detection reagent (Vector Laboratories, UK) according to the manufacturer's instructions. Sections (5 μm) were mounted and were imaged using an Olympus digital microscopy/slide scanning system (Olympus Soft Imaging Systems, Germany) with a x40 objective with dotSlide v2.2 software. Immunoreactivity was assessed in a blinded manner by a consultant histopathologist (MAK).

### Statistical analysis

For comparisons between groups, statistical significance (*p* < 0.05) was determined by unpaired *t*-test assuming unequal variances or by one-way or two-way ANOVA with Holm-Sidak multiple comparisons test using GraphPad Prism version 6.03 for Windows, GraphPad Software, La Jolla California USA. Drug combination indexes were calculated using Biosoft's CalcuSyn v2.11 [51]. Kaplan-Meier analysis was performed with IBM SPSS Statistics v22, while immunoblot densitometric analysis with Image J v1.48.
